# 

**Published:** 1921-04-23

**Authors:** 


### Personalia.

An interesting ceremony took place at the Rotunda Hospital, Dublin, on Wednesday last, when Sister Barker, who has been connected with the hospital for many years, was presented on behalf of past and present pupils with a gold wristlet-watch in appreciation of her long service and valuable and patient teaching.

Performances of the Japanese operetta, "Princess Juju," are to be given by the South Durham Street Mission choir in Sunderland next week, on the initiative of two well-known nurses in the city, Miss Beal and Miss Haswell, who have taken this mode of marking the close of their long labours for the sick. The entire proceeds will be handed over to the Royal Infirmary as their thankoffering—a gracious and unique mode of saying farewell.

### Points from Annual Reports.

### New Sunderland Hospital.

The Governors of Monkwearmouth and Southwick Hospital, Sunderland, have bought the Ashville Estate, on Newcastle Road, in order to build there a new hospital at a cost of £40,000. It will accommodate eighty beds in single-storeyed pavilions, and the object is to provide the increased accommodation so much needed at the hospital.

### Leicester Royal Infirmary.

The annual report contains 241 pages. A subscription list, totalling £9,520, accounts for many of these, and Hospital Saturday Fund yielded £19,760 to the hospital and £11,760 to the convalescent home. The total expenditure last year was £62,992 and the total income £59,773, but only £2,182 of the legacies received, £6,016, was carried to the general account.

### Royal Sussex County Hospital.

The annual report and accounts of this hospital should be carefully read, as we are reminded by the Secretary. It is true that there was an excess of income over expenditure in 1920, amounting to £17,053, but had a very large legacy not come in the balance would have been on the other side. Unfortunately, large legacies are not annual affairs, and moreover the hospital was saddled with a deficit of £5,025 from the previous year and is faced by an extremely urgent building programme to provide more nursing accommodation. Help for this, as well as for current expenses, is badly needed.

### Medical Appointments.

Mr. C. S. Neame, L.D.S., has been appointed school dentist to the County Borough of East Ham Education Committee. Mr. Neame, who studied at Guy's Hospital, was later dental house surgeon there.

Mr. I. R. Kay-Mouat, B.A., M.B., B.Ch., D.P.H., has been appointed by the Secretary of State for the Colonies to be Assistant Principal to the Medical School, Singapore. Mr. Kay-Mouat is a graduate of, and was demonstrator in pathology, at Bristol University.

### A Live Local Committee.

One of the organisations scattered over the London district which has contributed to the Hospital Saturday Fund's success, reported in our issue of April 16, is that of the Clapham, Balham, and Tooting Committee, the Chairman of which is Mr. G. A. Lewcock. For many years the Committee have aimed at collecting £1,000; this amount was exceeded in 1920 by nearly £500, showing an increase on the previous year of £578. This result is largely due to the local Committee's energetic efforts and excellent organisation, upon which Clapham, Balham, and Tooting are to be heartily congratulated.

### British Hospitals Association.

The second meeting of the North-Western Regional Committee of the British Hospitals Association was held at the Royal Infirmary, Manchester, on April 8. It was resolved that the Committee shall meet quarterly, or oftener if necessary, and that the meeting-place shall be the Manchester and Liverpool Royal Infirmaries alternately. Hospitals shall be invited to nominate two representatives each, the qualification for membership being governed by annual subscription to the British Hospitals Association. The following were appointed the Executive Committee:—Chairman: Sir Wm. Cobbett (Manchester Royal Infirmary); Mr. H. Wade Deacon (Liverpool Royal Infirmary), Capt. W. G. T. Currie (Chester Royal Infirmary), Mr. J. Gibson (Preston Royal Infirmary), Rev. J. E. Samuel (Blackburn Royal Infirmary), and Mr. E. F. Pearce (Stockport Infirmary), with Mr. Frank Hazell (Manchester Royal Infirmary) as Hon. Secretary.

It was resolved to ask the Council of the British Hospitals Association (1) to press the hospitals' claim to preferential treatment in respect of telephone service on the ground that a large proportion of the outward calls are in respect of public service; and (2) to take steps to bring to the notice of the Select Committee of the House of Commons on the Dangerous Drugs Act the position of the voluntary hospitals in this connection.

THE

### COLLEGE OF NURSING

(Limited by Guarantee.)

### What the Local Centres are Doing.

### LONDON CENTRE.

(Secretary, M. A. Bompas, 7 Henrietta Street, W. 1.)

Thursday, May 5, 8 p.m.—Annual General Meeting of members at the College of Ambulance, 56 Queen Anne Street, Cavendish Square, W. 1.

### CORNWALL CENTRE, TRURO.

(Hon. Secretary, Miss E. M. Jordan, Convalescent Homes, Perranporth.)

The annual meeting was held at the Royal Cornwall Infirmary on April 6, Miss Chaff, A.R.R.C., the President, in the chair. Disappointment was expressed at the unavoidable absence of Miss Sheriff MacGregor owing to the threatened railway strike. Miss Billing, R.R.C., Matron, Torbay Hospital, Torquay, spoke, and her address was much appreciated. Miss Chaff very kindly entertained the members to tea.

### NORTHUMBERLAND AND DURHAM CENTRE, NEWCASTLE-ON-TYNE.

(Secretary, Miss M. Toyne, 17 Windsor Terrace, Jesmond.)

The Annual Meeting, held at the Nurses' Club on Saturday, April 16, at 3 p.m., was well attended. Miss Brown was re-elected President, Miss Amour Hon. Treasurer, and Miss F. Jones Hon. Secretary, in succession to Miss M. Herbert, who has resigned. The financial statement submitted by the Hon. Treasurer shows a substantial balance in hand. The Secretary reported that the membership of the Centre now numbers 126. The Club has been much used by nurses for times varying from one night to two or three weeks.

### READING CENTRE.

At the meeting held at the Royal Berkshire Hospital, Reading, on April 16, it was decided to form a Local Centre. A meeting will be held on Saturday, April 30, at 3 p.m., to elect officers. Miss Wynne, who has consented to act as Hon. Secretary *pro tem.*, will be glad to receive names and addresses of all members wishing to join the Centre.

### SHEFFIELD CENTRE.

Annual Meeting and Election.

The year's report presented at the Annual General Meeting, held on April 12, shows there is an increase in membership, though the attendance at the fortnightly lectures has been poor. The finances are satisfactory, owing to the successful results of the Sale of Work and of the Dance held in January. Great regret is felt that Miss Smeeton, R.R.C., is not able to offer herself for re-election as Local Representative, on account of the pressure of her work as Matron of the Royal Infirmary and as Acting Matron for the 3rd Northern Hospital (T.F.). For the Committee vacancies it was resolved to nominate a sister from each hospital, and also from the different branches of the Municipal Nursing. Sixteen candidates were nominated for nine seats. The result of the election of officers and Committee is as follows: Local Representative, Miss Earle, R.R.C.; Hon. Secretary, Mrs. J. Baines; Hon. Treasurer, Mrs. C. E. R. Watson; Hon. Press Secretary, Miss C. E. Abbott; Members of Committee, Miss Smeeton, R.R.C., Miss Hollis (Matron, Children's Hospital), Miss Lewis (Matron, City's Infectious Hospital), Miss Hancocks (Superintendent Queen's Nurses, Sheffield), Miss Buckle (Superintendent Queen's Nurses, Rotherham), Miss Bray (Queen's Nurse), Sister Muir (Royal Hospital), Sister M. A. Watson (School Nurse), Mrs. McClean. It was agreed that next session the meetings shall be held at 7.30 p.m. instead of at 6 p.m. as before, and that the members should be asked to provide the programme on certain nights. It is hoped by these means to secure a better attendance and a greater interest in the Centre.

Forthcoming Meeting.

Monday, May 2, at 8 p.m.—At the Royal Hospital, Miss Sheriff MacGregor on "The Special Aims of the College."

### GLASGOW CENTRE.

(Hon. Secretary, Miss Moseley, Western District Hospital, Oakbank.)

The meeting arranged for Saturday, April 23, has been unavoidably postponed.

### ANNUAL SUBSCRIPTIONS TO CENTRES.

Centre members generally are reminded that their annual subscriptions are now due, and should be paid either at the annual meeting of their Centre or be sent by post to the Hon. Treasurer.

### Forthcoming Events.

Invalid Children's Aid Association.—Annual Meeting, Tuesday, April 26, 3 p.m., at 18 Carlton House Terrace, by kind permission of the Hon. J. J. and Lady Violet Astor, Lord Queenborough in the chair. Speakers, Miss Lena Ashwell, Father Bernard Vaughan, Sir Napier Burnett, Mr. Dennis Eadie, and others.

Children's Fresh Air Mission.—Annual Meeting, Staple Inn Hall, Holborn (by kind permission of the Institute of Actuaries), Tuesday, April 26, 3.30 p.m. The Marquess of Northampton will preside. Speakers, Lt.-Col. M. Archer-Shee, Lady Cooper, Col. Sir W. H. Dunn, Bart., and others. Tea will be served after the meeting.

Zenana Bible and Medical Mission.—Annual Meeting, Wednesday, April 27, 3 p.m., at the Central Hall, Westminster, Rt. Hon. Lord Meston, K.C.S.I., in the chair. Speakers, Miss G. A. Gollock, Mrs. Theodore Pennell, M.B., and Miss Janet Todd, of Kasur.

Derbyshire Royal Infirmary.—Half-yearly meeting of Governors in the Board Room at the Infirmary, Wednesday, April 27, at 2.15 p.m. Special business, to elect eight Governors to serve upon the Special Elective Committee; to rescind certain Rules. After the meeting Governors and friends are invited to attend the annual service by the Free Churches at St. Mary's Gate Baptist Church, Derby. The sermon will be preached on behalf of the funds of the charity by Rev. A. R. Henderson, M.A., D.D., at 4.30 p.m.

April 23, 1921] [Price Twopence

### The Hospital

**The College of Nursing (Limited by Guarantee)** 74

What the Local Centres are Doing.

**RATES OF SUBSCRIPTION** (post free, payable in advance).

Three months, 3/3; six months, 6/6; twelve months, 13/-. (Should the cost of printing and paper as well as increased postage, necessitate alteration of these rates, the period paid for will be reduced proportionately on unexpired subscriptions.) THE HOSPITAL "Index" to Volume LXVIII., April to September 1920, is now ready, price 1/1 per copy, post free. Address The Manager, THE HOSPITAL, "The Hospital" Building, 28/29 Southampton Street, Strand, London, W.C. 2.

### MANAGER'S NOTICES.

**All letters on business matters must be addressed to The Manager, "The Hospital," 28 and 29 Southampton Street, London, W.C.**

Rates for Subscription (payable in advance).

| | | | |
|---|---|---|---|
| Three Months (including Postage) | ... ... | 3s. | 3d. |
| Six Months do. | ... ... | 6s. | 6d. |
| Twelve Months do. | ... ... | 13s. | 0d. |

Subscriptions, which may commence at any time, are payable in advance. Cheques and Post-Office Orders should be crossed London County and Westminster Bank, Covent Garden Branch, and made payable to the Manager of The Hospital.

Should the cost of printing and paper, as well as increased postage, necessitate alteration of these rates, the period paid for will be reduced proportionately on unexpired subscriptions.

